# Self-reported Illness Experiences and Psychosocial Outcomes for Reservation-Area American Indian Youth During COVID-19

**DOI:** 10.1001/jamanetworkopen.2022.31764

**Published:** 2022-09-14

**Authors:** Linda R. Stanley, Meghan A. Crabtree, Randall C. Swaim, Mark A. Prince

**Affiliations:** 1 Colorado State University, Fort Collins

## Abstract

**Importance:**

Impacts of COVID-19 on reservation-area American Indian youth are unknown and may be substantial owing to the significant COVID-19 morbidity and mortality experienced by American Indian populations.

**Objective:**

To measure self-reported illness experiences and changes in psychosocial factors during the COVID-19 pandemic among reservation-area American Indian youth.

**Design, Setting, and Participants:**

This cross-sectional study included a random sample of US schools on or near US Indian reservations during Spring 2021, stratified by region, with students in grades 6 to 12 completing cross-sectional online surveys. All enrolled self-identifying American Indian students in grades 6 to 12 attending the 20 participating schools were eligible to be surveyed; participants represented 60.4% of eligible students in these schools. Data were analyzed from January 5 to July 15, 2022.

**Exposures:**

Onset of the COVID-19 pandemic.

**Main Outcomes and Measures:**

Outcomes of interest were COVID-19 self-reported illness outcomes for self and family and close friends; perceived changes in family and friend relationships, school engagement, social isolation, and other psychological factors since the COVID-19 pandemic began; and worry over COVID-19–related health outcomes.

**Results:**

A total of 2559 American Indian students (1201 [46.9%] male; 1284 [50.2%] female; 70 [2.7%] another gender; mean [SD] 14.7 [8.9] years) were included in the analysis. Approximately 14% of the sample reported having had a test result positive for SARS-CoV-2 infection (14.3% [95% CI, 11.4%-17.6%]), a higher rate than for all cases nationally at the time of the survey. Regarding prevalence of COVID-19 among family and close friends, 75.4% (95% CI, 68.8%-80.9%) of participants reported having at least 1 family member or friend who had contracted COVID-19, while 27.9% (95% CI, 18.8%-39.3%) of participants reported that at least 1 family member or close friend had died of COVID-19. Regarding psychosocial impacts, COVID-19 was associated with strained friend relationships (eg, 34.0% [95% CI, 28.4%-40.0%] of students reported worry over losing friends), lower school engagement, and less social connectedness (eg, 62.2% [95% CI, 56.7%-67.4%] of students reported feeling less socially connected to people), although more than 60% of students also reported feeling no change or a decrease in negative emotions. Males were less likely to report perceived negative impacts, especially for negative emotions such as sadness (29.2% [95% CI, 23.3%-35.9%] of males vs 46.1% [95% CI, 43.9%-48.3%] of females reported feeling more sad) and anxiety (21.8% [95% CI, 18.2%-25.8%] of males vs 39.2% [95% CI, 34.1%-44.6%] of females reported feeling more anxious).

**Conclusions and Relevance:**

This cross-sectional study provides novel insight into the perceived experiences of reservation-area American Indian youth, a population at uniquely elevated risk of poor health status and health care access, during the COVID-19 pandemic. Although mortality and morbidity rates from COVID-19 were high on American Indian reservations, student reports of psychosocial impacts were complex and suggest many students were resilient in the face of the pandemic. These findings could be used to understand and address the challenges facing American Indian youth due to the pandemic and to guide future research that examines the factors and processes associated with the reported outcomes.

## Key Points

**Question:**

How did American Indian youth who live on or near reservations experience the COVID-19 pandemic?

**Findings:**

This cross-sectional study among 2559 American Indian students in grades 6 through 12 found that approximately 14% of the sample reported having had a test result positive for SARS-CoV-2 infection, a higher rate than for all cases nationally and for children nationally, and three-quarters of the sample reported someone close contracting COVID-19, while more than one-quarter reported someone close dying from COVID-19. Regarding perceived psychosocial impacts, COVID-19 was associated with strained friend relationships, lower school engagement, and less social connectedness, although more than 60% of students reported feeling no change or a decrease in negative emotions, such as sadness and anxiety.

**Meaning:**

These findings suggest that although COVID-19 mortality and morbidity rates were high on American Indian reservations, psychosocial impacts were complex and many students were resilient in the face of the pandemic.

## Teaser

This cross-sectional study assesses self-reported illness experiences and changes in psychosocial factors during the COVID-19 pandemic among reservation-area American Indian youth.

## Introduction

COVID-19 has resulted in millions of deaths and disrupted the health, economic security, and well-being of people worldwide. While youth are at lower risk of morbidity and mortality from COVID-19,^1,2^ they have been subjected to many stressors, including school disruptions, extended home confinement, grief associated with morbidity and mortality of significant others, and uncertainty regarding their safety and security.^3^

A group at particularly high risk for COVID-19–related consequences are American Indian youth. American Indian individuals, in general, were at elevated risk for contracting COVID-19 and for experiencing more severe illness,^4,5^ and infection rates on some US Indian reservations were significantly greater than general population rates, with some case rates many times higher their home-state rate or the national rate.^6,7^ By March 3, 2021, American Indian and Alaska Native individuals had the highest COVID-19 mortality rates in the US, with age-adjusted rates showing American Indian or Alaska Native individuals 3.3-fold more likely to die than White individuals.^8^ Additionally, American Indian or Alaska Native individuals contracted COVID-19 at younger ages than their White counterparts,^9^ and American Indian individuals who died from COVID-19 were generally younger than White individuals.^5^ Prior to the pandemic, American Indian individuals were at substantially increased risk for depression, generalized anxiety,^10^ and suicide^11^ compared with the general population.

### COVID-19–Related Social Impacts

Stay-at-home orders and schools using online teaching changed the social world of youths dramatically. These changes may have positive and negative effects, especially with the developmental shift in the social environment from parents to peers. Increased time with family can be beneficial; however, for households with high levels of stress, additional time with family can exacerbate conflict and psychological distress.^12^ Mandated lockdowns and social distancing policies limited youths’ opportunities to maintain face-to-face contact with peers, increasing risk for mental health consequences.^13^ While technology may offset impacts of reduced face-to-face contact, youths may experience social isolation owing to physical separation from others.^14^ Moreover, school closures may create serious adverse consequences, especially for those attending reservation-area schools, by reducing access to mental and physical health resources.^15,16,17^ Additionally, academic engagement may decrease for some students,^18^ while for others, engagement may improve with decreased social pressures in the school setting.^19^

### COVID-19–Related Mental Health Impacts

Two systematic reviews found that youth reported higher rates of anxiety, depression, and stress due to the pandemic.^3,20^ A meta-analysis of 29 studies of anxiety and depression symptoms in youth (age ≤18 y) using data gathered in 2020 found elevated depression and elevated anxiety symptoms.^21^ Furthermore, higher prevalence of anxiety and depression symptoms occurred in females and in studies occurring later in 2020, while higher depression symptoms occurred in older youth.^21^ A national US poll of parents of youths aged 13 to 18 years conducted in January 2021 found similarly high parent-reported rates of depression and anxiety.^22^

This study presents self-reported perceived impacts of COVID-19 on American Indian youth who live on or near reservations (hereafter, *reservation-area*). The Our Youth, Our Future (OYOF) study^23^ annually collects nationally representative substance use data for reservation-area middle and high school students. During Spring 2021, COVID-19–related measures were added to the survey; this study presents findings.

## Methods

All procedures for this cross-sectional study were approved by the Colorado State University institutional review board and by appropriate tribal research review boards, school boards, and school staff. Because data are anonymous and cannot be linked to any individual, a passive consent process was used, whereby parents or guardians were informed of the survey and could opt out their child. Youth provided assent at the start of the survey. This study follows the Strengthening the Reporting of Observational Studies in Epidemiology (STROBE) reporting guideline.

### Sample

A description of the sampling frame, sample, and recruitment procedures, and survey procedures is presented in the eMethods in the Supplement. Study data were collected from 20 schools (17.1% of 117 schools recruited) that participated in the OYOF study during Spring 2021 (eMethods in the Supplement). The regional distribution of schools was 3 schools (15%) in the Northern Plains, 1 school (5%) in the Northwest, 4 schools (20%) in the Upper Great Lakes, 2 schools (10%) in the Northeast, 4 schools (20%) in the Southern Great Plains, 1 school (5%) in the Southeast, and 5 schools (25%) in the Southwest. All schools were public, with the percentage of American Indians enrolled varying between 20% and 100% (mean, 58.7%), and all schools had at least some days of in-person instruction. Compared with nonparticipating schools, participating schools had fewer students receiving free lunch and fewer American Indian students enrolled. Specific identities of tribes and reservations are kept confidential. eTable 1 in the Supplement provides further information on characteristics of participating schools compared with recruited schools that did not participate. All enrolled students in grades 6 through 12 attending the participating schools were eligible to be surveyed.

### Procedures

Before survey administration, parents could opt their child out of the survey through a passive consent process (<1% of parents opted out). Surveys were administered online with Qualtrics software to all 6th through 12th grade students enrolled and attending school (in person or virtually) on the survey dates. Among American Indian students, 59.8% reported taking the survey at school, while 36.0% reported taking it at some other location (4.2% did not respond to this question). Responses were anonymous, and students were instructed to skip questions they did not wish to answer.

### Measures

We used measures from the COVID-19 OBSSR Research Tools instruments,^24^ in addition to creating items to measure specific risk and protective factors that may be affected by COVID-19. The following categories of measures were included: COVID-19 self-reported illness experiences for self and family and close friends; perceived changes in family and friend relationships, school engagement, social isolation, and psychological factors; and worry over COVID-19–related health outcomes. Further details on categories are provided in the eMethods in the Supplement.

### Planned Missingness

Owing to the large number of items on the OYOF survey, a planned missing data design was used.^25^ After completing core items (eg, demographics and COVID-19 illness experience measures), each participant randomly received 1 of 3 samples of measures, with approximately two-thirds of respondents receiving any item. In addition, blocks of items (excluding core items) were randomly ordered. Planned missing data are considered missing completely at random, and randomization of order reduced the chance of systematic missingness.^26^

### Statistical Analysis

For each item, frequency of outcomes (with 95% CIs) were computed using survey commands of Stata statistical software version 15.1 (StataCorp), designating stratification by region and school as the primary sampling unit. Observations were weighted to correct for overrepresentation or underrepresentation by region, with weights based on US Census reservation populations for ages 10 to 19 years (eMethods in the Supplement). Nonplanned missingness varied between 2.3% and 24.3% for individual items, with lower rates for illness experiences and higher rates for psychosocial factors (which appeared later in the survey).

Differences by grade group (grades 6-8 vs 9-12) and gender were tested using Pearson χ^2^ tests adjusted for complex samples.^27^ Because of few differences by grade, results are reported for all grades combined. Significant differences by gender for psychosocial variables were found; thus, these results are reported by gender, with categories of female, male, and another gender. Finally, pairwise correlations between illness experiences and self-reported changes in psychological factors (with 95% CIs) were calculated.

*P* values were 2-tailed, and statistical significance was set at *P* = .05. Data were analyzed from January 5 to July 14, 2022.

## Results

### Participants

A total of 2559 students (1201 [46.9%] male; 1284 [50.2%] female; 70 [2.7%] another gender; mean [SD] age, 14.7 [8.9] years) in grades 6 through 12 self-identifying as American Indian were included. A total of 1574 respondents (61.5%) reported American Indian as their only race and ethnicity, while 985 participants (38.5%) reported an additional race or ethnicity. Sample sizes, mean age, and gender by grade race and ethnicity and regional distribution of students are provided in eTable 2 in the Supplement.

### COVID-19 Illness Experiences

Table 1 presents participants’ perceived COVID-19 illness experiences among themselves and family and close friends. No differences by gender were found for these items; thus, combined results are presented. For surveyed students, 19.0% (95% CI, 15.6%-22.9%) of participants reported having had COVID-19, and of these, 75.2% (95% CI, 65.7%-82.7%) reported a positive test result. Approximately 61% of participants reported having been tested for COVID-19 (61.1% [95% CI, 54.4%-67.4%] of students), with 23.3% (95% CI, 19.7%-27.4%) of participants reporting having had a positive test result. Of participants who reported having had COVID-19, 45.4% (95% CI, 40.6%-50.3%) reported that they did not feel sick.

**Table 1.** American Indian Students Self-reporting COVID-19 Illness Experiences for Self and Others

Question	% (95% CI) (N = 2499)
Have you had COVID-19?	
Yes	19.0 (15.6-22.9)
I tested positive for COVID-19^a^	75.2 (65.7-82.7)
I wasn’t tested for COVID-19^a^	24.8 (17.3-34.3)
No	81.0 (77.8-84.4)
I tested negative^b^	57.8 (50.8-64.5)
I’ve never been tested^b^	42.2 (35.5-49.2)
Have you been tested for COVID-19?^c^	
Yes	61.1 (54.4-67.4)
Tested positive	23.3 (19.7-27.4)
No	38.9 (32.6-45.6)
How sick did you get? (n = 438)	
I didn’t feel sick	45.4 (40.6-50.3)
I felt sick, but I didn’t see a doctor	35.1 (30.5-40.0)
I felt sick and had to see a doctor	17.4 (11.4-25.7)
I felt sick and was in a clinic/hospital for at least one night	2.0 (1.2-3.4)
How many of your family members or close friends have had COVID-19? (n = 2475)^d^	
0	24.7 (19.1-31.2)
≥1	75.3 (68.8-80.9)
1	14.4 (12.1-17.0)
2-3	31.2 (28.1-34.4)
≥4	29.8 (24.6-35.5)
How many of your family members or close friends stayed in the hospital because of COVID-19? (N = 2476)	
0	62.5 (56.0-68.5)
≥1	37.5 (31.5-44.0)
1	19.2 (17.5-21.1)
2-3	13.6 (9.6-18.9)
≥4	4.7 (2.9-7.4)
How many of your family members or close friends have died from COVID-19? (N = 2467)	
0	72.1 (61.7-81.2)
≥1	27.9 (18.8-39.3)
1	12.7 (9.0-17.6)
2-3	10.0 (6.6-14.9)
≥4	5.3 (2.8-9.8)

^a^ Results reflect the percentage out of those responding that they had had COVID-19.

^b^ Results reflect the percentage out of those responding that they had not had COVID-19.

^c^ Total number tested was computed by combining categories “I tested positive for COVID-19” and “I tested negative for COVID-19.”

^d^ Distribution differs between grades 6 to 8 and grades 9 to 12, with 69.4% (95% CI, 60.8-76.8) of 6th to 8th grade students reporting a family member or close friend having had COVID-19, compared with 79.3% (95% CI, 73.7, 83.9) of 9th to 12th grade students (*P* = .04).

Regarding prevalence of COVID-19 among family and close friends, 75.4% (95% CI, 68.8%-80.9%) of participants reported having at least 1 family member or friend who had COVID-19, while 29.8% (95% CI, 24.6%-35.5%) of participants reported having 4 or more family members or friends with COVID-19. Of the total sample, 37.5% (95% CI, 31.5%-44.0%) of participants reported at least 1 family member or close friend had been hospitalized, and 27.9% (95% CI, 18.8%-39.3%) of participants reported that at least 1 family member or close friend had died of COVID-19.

### Changes Related to Social Factors

There were few differences in perceived changes by gender in relationships with family and friends, school factors, and social connectedness; therefore, combined results are presented in Table 2. Point differences among genders are provided in eTable 3 in the Supplement.

**Table 2.** American Indian Students Reporting COVID-19–Related Perceived Impacts on Friends, Family, School, and Self, by Gender^a^

Dimension	No.	Students reporting somewhat or very true, % (95% CI)		
		Female	Male	Another gender
Impacts on family relationships				
I spend more time with my family	1479	52.6 (48.6-56.5)	54.6 (48.0-61.1)	45.3 (23.1-69.6)
My family has more conflict	1448	29.9 (24.4-34.8)	28.3 (24.5-32.5)	35.3 (17.4-58.7)
My family is closer	1463	40.1 (35.3-46.7)	41.9 (35.6-48.4)	29.9 (10.8-60.0)
My parents/guardians supervise my activities more	1453	36.7 (31.0-42.8)	32.9 (24.9-41.9)	43.3 (17.6-73.1)
Impacts on relationships with friends				
I see my friends more remotely or online (texting, social media)	1475	51.2 (42.5-59.8)	45.8 (40.7-50.9)	58.8 (36.0-78.3)
I see my friends more in person	1480	27.2 (23.2-31.6)^b^	34.5 (28.6-40.9)	24.0 (11.9-42.5)
I’m worried that I will lose friends because I can’t see them as often	1475	37.2 (31.0-44.0)^c^^,^^d^	29.5 (24.0-35.8)^e^	58.9 (44.7-71.7)
I feel closer to my friends	1490	24.7 (22.1-27.4)^c^	34.4 (29.8-39.3)	32.7 (20.7-47.5)
Impacts on school				
I am getting better grades in school	1457	27.2 (23.3-31.5)^c^	33.8 (23.1-38.8)	26.9 (9.4-56.7)
I am enjoying school more	1475	24.0 (18.4-30.6)	30.3 (25.3-35.8)	36.8 (26.3-48.7)
It is harder for me to focus on my schoolwork	1474	60.1 (52.9-66.8)^b^	48.1 (42.5-53.7)^f^	74.4 (50.7-89.2)
I am falling behind in my schoolwork more	1458	54.1 (45.1-62.7)^b^	42.7 (36.8-48.9)^e^	67.0 (49.0-81.0)
I attend school more (either remotely or in person)	1456	43.9 (36.2-51.9)	42.9 (36.8-49.2)	41.6 (27.9-56.6)
I am more worried about school	1223	55.3 (47.0-63.4)^c^	46.7 (40.1-53.4)^f^	63.7 (43.7-79.8)
I miss participating in or attending school events	1465	57.8 (51.4-63.9)^c^	45.8 (38.2-53.6)	55.4 (34.1-74.9)
I don’t have the technology I need at home to complete my schoolwork (computer, wifi/internet)	1438	18.2 (14.6-22.4)	16.4 (12.8-20.7)	16.2 (4.6-43.9)
I don’t have the space I need at home to complete my schoolwork (a desk to work on, a quiet room)	1435	36.1 (28.7-44.2)^c^	26.2 (23.0-29.6)^e^	47.4 (33.3-61.9)
It is difficult to concentrate on my schoolwork because of what else is happening at home	1434	41.3 (51.8-65.3)	39.0 (33.1-45.2)	51.7 (37.0-66.2)
Impacts on social connection				
I spend more time alone	1453	59.1 (54.0-64.0)^c^^,^^d^	43.9 (40.2-47.6)^e^	89.6 (74.2-96.3)
I feel less socially connected to people^g^	2456	68.7 (63.6-73.4)^c^	55.6 (49.8-61.1)	59.5 (46.4-71.4)

^a^ Questions are presented verbatim as they appeared in the survey. Sample sizes for all but the question regarding social connectedness are significantly less than 2559 partially owing to the planned missingness design and partially owing to students not responding to the question. For all items but social connectedness, the sample sizes by gender were a mean (SD) of 722 (36) females, 668 (37) males, and 39 (1) individuals identifying as another gender.

^b^ Female and male estimates differ at *P* < .05.

^c^ Female and male estimates differ at *P* < .01.

^d^ Female and another gender estimates differ at *P* < .01.

^e^ Male and another gender estimates differ at *P* < .01.

^f^ Male and another gender estimates differ at *P* < .05.

^g^ This question was based on a 5-point scale of much less socially connected, less socially connected, about the same, more socially connected, and much more socially connected. Percentages indicate those who answered they felt much less or less socially connected. The larger sample size is due to this question not being part of the planned missing design so all students could answer it. Also, it was near the beginning of the survey and thus had a lower percentage of missingness due to survey attrition.

#### Family Relationships

Approximately half of students (53.3% [95% CI, 49.0%-57.6%]) reported spending more time with family. While 29.4% (95% CI, 25.8%-33.2%) of students reported more family conflict, 41.1% (95% CI, 37.4%-45.0%) of students reported their family was closer during the pandemic. Approximately one-third of students (35.0% [95% CI, 28.5%-42.0%]) also reported that parents or guardians supervised their activities more (Table 2). No significant differences by gender were found.

#### Friend Relationships

Nearly half of all students reported seeing friends more often remotely or online (48.7% [95% CI, 43.2%-54.2%] of students) while 30.6% (95% CI, 26.3%-35.3%) of students reported seeing friends more often in-person, with males more likely to report seeing friends in-person than females (Table 2). Worry over losing friends (34.0% [95% CI, 28.4%-40.0%] of students) was more likely to be reported by students identifying as another gender (58.9% [95% CI, 44.7%-71.7%] of students) than by females (37.2% [95% CI, 31.0%-44.0%] of female students) or males (29.5% [95% CI, 24.0%-35.8%] of male students). Finally, less than one-third of students reported feeling closer to their friends (29.4% [95% CI, 26.4%-32.7%] of students), with females least likely to report greater closeness (Table 2).

#### Changes Related to School

Generally, students were more likely to report negative changes related to school than positive ones. Approximately 30% of students reported getting better grades (30.4% [95% CI, 26.7%-34.3%] of students) or enjoying school more (27.2% [95% CI, 23.4%-31.5%] of students) during the pandemic, while approximately one-half reported having a harder time focusing on schoolwork (54.7% [95% CI, 49.9%-59.5%] of students), falling behind on schoolwork (48.9% [95% CI, 43.9%-53.9%] of students), worrying about school (51.5% [95% CI, 44.5%-58.5%] of students), and missing participation in school events (52.0% [95% CI, 45.2%-58.8%] of students), with significantly more nonmale students reporting these (Table 2). Finally, less than 20% of students (17.2% [95% CI, 14.3%-20.7%] of students) reported not having the technology for schoolwork, while 31.6% (95% CI, 26.9%-36.7%) of students reported not having space to complete schoolwork, and 40.5% (95% CI, 35.4%-45.8%) of students reported difficulty concentrating on schoolwork because of what was happening at home.

#### Changes Related to Social Connection

Most students reported spending more time alone (52.6% [95% CI, 49.5%-55.6%] of students) and feeling less socially connected to people (62.2% [95% CI, 56.7%-67.4%] of students). More female students reported these changes than males did, and more students who identified as another gender reported spending more time alone than other students (Table 2).

### Psychological Measures

Table 3 presents worry over morbidity and mortality by gender. Point differences among genders are presented in eTable 4 in the Supplement. More females reported worry than males. More than 70% of students expressed worry over a family member getting COVID-19 (71.3% [95% CI, 63.8%-77.7%] of students) or dying from COVID-19 (82.4% [95% CI, 77.1%-86.8%] of students), compared with 47.7% (95% CI, 39.2%-56.4%) of students who worried about getting COVID-19 and 60.1% (95% CI, 54.2%-65.7%) of students dying from COVID-19 themselves. Most students were also concerned about giving someone COVID-19 (62.8% [95% CI, 54.8%-70.2%] of students).

**Table 3.** American Indian Students Reporting Being Worried or Very Worried Over Potential COVID-19 Morbidity and Mortality Outcomes, by Gender^a^

How worried are you about…	No.	Students, % (95% CI)		
		Female	Male	Another gender
…getting COVID-19?	1538	50.6 (44.1-57.0)^b^	45.1 (33.1-57.7)	42.4 (33.2-52.2)
…a family member getting COVID-19?	1559	78.8 (69.7-85.6)^c^	64.0 (56.5-70.7)	66.0 (47.8-80.4)
…dying from COVID-19?	1528	65.6 (57.7-72.8)^d^^,^^e^	55.7 (48.6-62.4)^f^	36.3 (26.1-47.9)
…a family member dying from COVID-19?	1528	86.4 (78.8-91.5)^d^	78.8 (73.5-83.2)	77.4 (57.0-89.9)
…giving someone else COVID-19?	1556	68.4 (59.6-76.0)^c^	57.4 (48.9-65.4)	59.7 (45.6-70.8)

^a^ Questions are presented verbatim as they appeared in the survey. Sample sizes in this table are significantly less than 2559 partially owing to the planned missingness design and partially owing to students not responding to the question. For all questions, the sample sizes by gender were a mean (SD) of 785 (9) females, 719 (10) males, and 39 (<1) individuals identifying as another gender.

^b^ Female and another gender estimates differ at *P* < .05.

^c^ Female and male estimates differ at *P* < .01.

^d^ Female and male estimates differ at *P* < .05.

^e^ Female and another gender estimates differ at *P* < .01.

^f^ Male and another gender estimates differ at *P* < .01.

Table 4 presents results by gender for perceived changes in psychological factors since COVID-19 started (95% CI), with responses grouped by whether the psychological factor was experienced less or much less, the same, or more or much more. Point differences (95% CI) among genders are presented in eTable 5 in the Supplement. Across all measures, approximately 30% to 40% of females and males reported no change in these factors, with no significant differences by gender. Conversely, a significantly larger percentage of males, compared with females, reported less negative affect and experiences since COVID-19 began for all measures, while females were significantly more likely to report more negative affect and experiences compared with males. For example, 43.5% (95% CI, 36.0%-51.3%) of males and 28.8% (95% CI, 25.6%-32.3%) of females felt less depressed, while 24.4% (95% CI, 20.9%-28.3%) of males and 40.2% (95% CI, 36.1%-44.4%) of females felt more depressed. Although more males and females reported no change or a decrease in negative emotions compared with those who reported an increase in negative emotions, it should be noted that more one-third of youth still reported experiencing these negative emotions often to very often (eTable 6 in the Supplement).

**Table 4.** American Indian Students Responding About Perceived Changes in COVID-19–Related Psychological Measures, by Gender^a^

Compared to before COVID-19, are you…	Students , % (95% CI)										
	Feeling more or much more than before COVID-19				Feeling about the same as before COVID-19				Feeling less or much less than before COVID-19		
	Female	Male	Another gender	Female		Male	Another gender	Female		Male	Another gender
More or less sad?	46.1 (43.9-48.3)^b^	29.2 (23.3-35.9)^c^	69.0 (35.5-90.0)	31.0 (26.3-36.0)		32.9 (26.6-39.9)	23.7 (8.5-51.1)	22.9 (19.3-27.0)^b^^,^^d^		37.9 (29.6-47.0)^c^	7.3 (1.6-28.1)
More or less lonely?	43.0 (39.0-47.1)^b^	28.3 (22.5-34.9)^e^	62.4 (35.5-83.3)	31.7 (27.0-36.8)		32.6 (25.7-40.3)	28.1 (14.8-46.8)	25.3 (23.6-27.1)^b^		39.1 (31.6-47.1)^c^	9.5 (1.0-51.3)
More or less depressed?	40.2 (36.1-44.4)^b^^,^^d^	24.4 (20.9-28.3^c^	61.0 (42.4-76.9)	31.0 (26.1-36.4)		32.1 (26.1-38.7)	32.0 (18.9-48.9)	28.8 (25.6-32.3)^b^^,^^f^		43.5 (36.0-51.3)^c^	6.9 (1.6-25.8)
More or less angry?^g^	37.2 (33.2-41.5)^b^	21.8 (15.8-29.3)^h^^,^^e^	44.6 (26.2-64.6)	38.3 (33.1-43.7)		38.4 (27.9-50.2)	34.9 (17.3-57.8)	24.5 (19.8-29.8)^b^		39.8 (32.4-47.6)^c^	20.5 (9.6-38.4)
More or less worried?	39.3 (34.2-44.7)^b^	29.5 (24.5-35.2)^c^	47.4 (35.8-59.3)	40.1 (35.1-45.2)		36.5 (32.7-40.3)	35.2 (21.1-52.6)	20.7 (16.4-25.7)^b^		34.0 (29.4-38.9)^c^	17.4 (7.9-33.9)
More or less anxious?	39.2 (34.1-44.6)^b^^,^^f^	21.8 (18.2-25.8)^c^	65.1 (48.0-79.0)	36.7 (32.6-41.1)		36.9 (31.3-42.9)	28.0 (16.8-43.0)	24.0 (20.4-28.1)^b^^,^^f^		41.3 (36.0-46.8)^c^	6.9 (1.6-24.8)
Having more or less trouble sleeping?^g^	44.8 (39.8-49.9)^b^^,^^f^	31.1 (27.0-35.6)^c^	69.3 (55.8-80.2)	34.7 (30.8-38.8)		35.2 (28.5-42.5)	28.3 (18.4-40.8)	20.5 (16.1-25.7)^f^^,^^h^		33.7 (27.4-40.7)^c^	2.4 (2.0-23.3)
More or less interested in normal activities?	38.1 (34.4-42.0)^d^	34.3 (29.6-39.3)^e^	62.4 (36.1-83.0)	39.1 (33.5-45.0)		34.2 (27.1-42.0)	17.0 (7.3-34.6)	22.8^h^ (19.5-26.5)		31.5 (24.3-39.8)	20.6 (9.4-39.3)
Having more or less trouble concentrating?	44.8 (40.3-49.4)^b^^,^^d^	35.4 (31.0-40.1)^c^	65.3 (48.5-79.1)	36.6 (32.1-41.2)		40.2 (32.5-48.4)	26.8 (14.4-44.3)	18.6(15.1-22.7)^b^^,^^d^^,^^h^		24.4 (18.6-31.4)^c^	7.9 (2.5-22.5)

^a^ Questions are presented verbatim as they appeared in the survey. Number of observations for each item varied from 1503 to 1534. Sample sizes are significantly less than 2559 partially owing to the planned missingness design and partially owing to students not responding to the question. For all questions, the sample sizes by gender were a mean (SD) of 767 (2) females, 701 (5) males, and 39 (1) individuals identifying another gender.

^b^ Female and male estimates differ at *P* < .01.

^c^ Male and another gender estimates differ at *P* < .01.

^d^ Female and another gender estimates differ at *P* < .05.

^e^ Male and another gender estimates differ at *P* < .05.

^f^ Female and another gender estimates differ at *P* < .01.

^g^ Students in grades 6 to 8 were more likely to report feeling more or much more this way. For students in grades 6 to 8, 33.1% (95% CI, 30.2%-36.1%) felt more angry and 43.5% (95% CI, 38.6%-48.5%) had more trouble sleeping. For students in grades 9 to 12, 27.7% (95% CI, 25.5%-30.0%) felt more angry and 34.9% (95% CI, 29.8%-40.5%) had more trouble sleeping.

^h^ Female and male estimates differ at *P* < .05.

Table 5 presents pairwise correlations between illness experiences and negative affect. Overall, correlations were relatively small (*r* < .13), though many were significantly different from zero and indicate a positive association between negative affect and number of illness experiences of family and friends. Fewer correlations of negative affect with hospitalization or death were significantly different from zero compared with those between negative affect and family or friends having COVID-19.

**Table 5.** Pairwise Correlations Between Illness Experiences and Self-reported Changes in COVID-19–Related Psychological Measures for American Indian Students^a^

Compared to before COVID-19, are you…	Correlation with No. of family/friends, *r* (95% CI)		
	With COVID-19	Hospitalized with COVID-19	Died from COVID-19
More or less sad?	0.133 (0.090 to 0.176)^b^	0.066 (−0.007 to 0.139)^b^	0.088 (0.008 to 0.168)^c^
More or less lonely?	0.086 (0.023 to 0.149)^b^	0.061 (−0.017 to 0.139)	0.061 (−0.023 to 0.145)
More or less depressed?	0.105 (0.058 to 0.152)^b^	0.059 (−0.012 to 0.130)	.052 (−0.052 to 0.156)
More or less angry?	0.103 (0.038 to 0.168)^b^	0.098 (0.025 to 0.171)^b^	0.084 (0.021 to 0.147)^b^
More or less worried?	0.079 (0.018 to 0.140)^c^	0.058 (−0.005 to 0.121)^c^	0.042 (−0.036 to 0.120)
More or less anxious?	0.106 (0.055 to 0.157)^b^	0.099 (0.056 to 0.142)^b^	0.053 (0.014 to 0.092)^b^

^a^ Questions are presented verbatim as they appeared in the survey. A positive correlation indicates a positive association between feeling more negative affect and the number of family and friends who had the illness experience.

^b^ *P* < .01.

^c^ *P* < .05.

## Discussion

### Illness Experiences

The findings of this cross-sectional study are consistent with prior works reporting high rates of SARS-CoV-2 infection and mortality among American Indian individuals. Approximately 14% of participants reported having received test results positive for SARS-CoV-2 infection, a higher rate than for all cases nationally (approximately 9.6% as of April 30, 2021) and for children nationally (4.8% as of April 29, 2021) at a similar time.^28^ Three-quarters reported someone close contracting COVID-19, while more than one-quarter reported someone close dying. A 2021 study^29^ found that American Indian or Alaska Native children account for up to 55% of children who lost a parent or other primary caregiver to COVID-19 and were 4.5-fold more likely to lose a parent or grandparent caregiver than White children. Numerous social risk factors tied to colonialism, historical trauma, and systemic discrimination have contributed to the disproportionate impact of COVID-19 in American Indian communities,^30,31^ including poorly funded public health infrastructure, limited medical facilities serving remote areas, prevalent underlying medical conditions, inadequate housing, and disproportionate representation in high-risk essential employment.^6,32,33,34^

Despite these challenges, most survey respondents reported receiving a COVID-19 test, and as of December 2021, the Centers for Disease Control and Prevention^35^ reported vaccination rates for American Indians to be the highest of all US racial or ethnic groups. These behaviors reflect the swift action of tribal nations to protect their communities, including strong mobilization efforts, utilizing existing vaccination programs, and imbuing cultural values into vaccination efforts.^36^ Despite these efforts, our findings suggest COVD-19–related exacerbation of burdens that American Indian youth experienced prior to the pandemic.^37,38^

### Perceived Psychosocial Impacts

Regarding perceived social impacts, our findings suggest that COVID-19 most negatively affected school engagement and feelings of social connectedness. Regarding school engagement, less than one-third of American Indian youth reported enjoying school more or getting better grades, while approximately one-half reported falling behind on schoolwork, difficulty focusing on schoolwork, worrying about school, and missing attending school events. Although the impact of COVID-19 on school engagement has been felt across the nation, the scope of the impact is not equivalent across schools. Students attending schools serving low–socioeconomic status areas, rural areas, and communities where most individuals are not non-Hispanic White have been uniquely impacted by school closures^39^; however, studies targeting reservation-serving schools are scarce to nonexistent. The prevalence of social isolation is particularly concerning, as greater social support is associated with mitigating mental health problems and psychological distress among youth experiencing adversity.^40^ Thus, robust inferential studies are needed to better understand the associations of COVID-19 with school engagement and connectedness in this population.

Importantly, a larger percentage of American Indian youth reported greater family closeness than reported greater conflict during the pandemic. For those youth, family closeness may be a source of resilience, as research has shown that close-knit families are associated with strong protection against psychological and behavioral health issues for American Indian youth.^41,42,43^ Yet, the percentage of American Indian youth reporting greater family conflict was notable, and recent studies, such as a 2021 study by Campione-Barr et al,^44^ have found negative interactions with family members to be associated with higher levels of general and COVID-19–specific psychological distress, particularly for youth experiencing greater isolation from friends.

The complexity of COVID-19–related impacts on youth mental health was illustrated in perceived changes in psychological factors since COVID-19 began. Negative affect and distress were most prevalent among nonmale youth, a finding consistent with other studies of youth mental health during COVID-19.^45,46,47^ Generally, more than one-third of female students and students self-identifying as another gender reported feeling more negative affect, while more than one-third of males reported feeling less negative affect. Also of note is the approximately one-third of students, regardless of gender, who reported no changes in negative affect. Thus, while a significant proportion of respondents, especially nonmales, reported feeling worse since COVID-19 started, more than 60% of respondents reported positive or no changes in negative affect. These latter results may reflect individual, family, and community resources that increased resilience, including tribal strengths, such as connectedness, family kinship, and collectivism, that were reported to be instrumental in tribal communities working toward a common goal of protecting their communities.^48^ In addition, decreased stress from day-to-day routines and in-person school may have attenuated negative affect for some students, especially males.^49^ Conversely, more illness among family and close friends may have led to increased negative affect. However, correlations between these variables were relatively small, suggesting this may not be a large factor. Finally, reported rates of negative affect were higher than those found in a national poll and in a meta-analysis of studies from across the world.^21,22^ In part, these higher rates reflect prepandemic mental health disparities.

### Limitations

While this study fills an important gap in the broader literature examining the perceived impacts of the COVID-19 pandemic among reservation-area American Indian youth, findings should be considered in light of several limitations. First, OYOF data are cross-sectional and rely on student self-reports. Furthermore, although the sampling method targeted a nationally representative sample of American Indian youth attending reservation-serving schools, substantial changes in school operating conditions in Spring 2021 made school recruitment and participation challenging, and participating schools may be better-resourced than nonparticipating schools. Participating schools were more likely to be public, off-reservation schools, have fewer students receiving free lunch, and have fewer American Indian students enrolled compared with nonparticipating schools. This may account for the relatively low percentage of students (<20%) who reported not having the technology to complete schoolwork. Similarly, attendance rates were at unprecedented lows for surveyed schools; consequently, students attending school regularly were more likely to be surveyed. Thus, these findings may underestimate perceived impacts of COVID-19 among American Indian youth. Additionally, the number of students identifying as another gender was small; thus, CIs for this group are large.

## Conclusions

The findings of this cross-sectional study provide novel empirical insight regarding the experiences of reservation-area American Indian youth, a uniquely vulnerable population, during the COVID-19 pandemic. Findings indicate that the perceived impacts of the COVID-19 pandemic among this population were complex. It is worthwhile to examine how various factors, such as family morbidity and mortality, contributed to the self-reported impacts presented in this study. Given major gaps in reporting of COVID-19 pandemic impacts among Indigenous communities globally,^50^ these findings, although descriptive, lay a foundation for better understanding through further research the COVID-19–related issues facing American Indian youth.

## Notes

Supplement.
eMethods.
**eTable 1.** Comparison of Participating Schools and Nonparticipating Schools in Spring 2021 Sample, By Type Of School, Location, Ethnicity Composition, and Percentage of Students Receiving Free Lunch **eTable 2.** Students By Grade and Gender, Mean Age By Grade, Race and Ethnicity, and Region **eTable 3.** Point Differences Among Genders for American Indian Students Reporting COVID-19–Related Perceived Impacts on Friends, Family, School, and Self **eTable 4.** Point Differences Among Genders in American Indian Students Responding Being Worried or Very Worried Over Potential COVID-19 Morbidity and Mortality Outcomes **eTable 5.** Differences by Among in American Indian Students Responding About Perceived Changes in COVID-19–Related Psychological Measures **eTable 6.** American Indian Students Responding on COVID-19–Related Psychological Measures, by Gender
eReferences.
