# Supplementary material for: Self-reported Illness Experiences and Psychosocial Outcomes for Reservation-Area American Indian Youth During COVID-19
Source: JAMA Netw Open. 2022 Sep 14;5(9):e2231764. doi: 10.1001/jamanetworkopen.2022.31764 (PMC9475383; doi:10.1001/jamanetworkopen.2022.31764)
Supplement: Supplement. — eMethods. eTable 1. Comparison of Participating Schools and Nonparticipating Schools in Spring 2021 Sample, By Type Of School, Location, Ethnicity Composition, and Percentage of Students Receiving Free Lunch eTable 2. Students By Grade and Gender, Mean Age By Grade, Race and Ethnicity, and Region eTable 3. Point Differences Among Genders for American Indian Students Reporting COVID-19–Related Perceived Impacts on Friends, Family, School, and Self eTable 4. Point Differences Among Genders in American Indian Students Responding Being Worried or Very Worried Over Potential COVID-19 Morbidity and Mortality Outcomes eTable 5. Differences by Among in American Indian Students Responding About Perceived Changes in COVID-19–Related Psychological Measures eTable 6. American Indian Students Responding on COVID-19–Related Psychological Measures, by Gender eReferences. [file jamanetwopen-e2231764-s001.pdf]

Supplemental Online Content

Stanley LR, Crabtree MA, Swaim RC, Prince MA. Self-reported illness experiences and psychosocial outcomes for reservation-area American Indian youth during COVID-19. *JAMA Netw Open*. 2022;5(9):e2231764. doi:10.1001/jamanetworkopen.2022.31764

eMethods.

eTable 1. Comparison of Participating Schools and Nonparticipating Schools in Spring 2021 Sample, By Type Of School, Location, Ethnicity Composition, and Percentage of Students Receiving Free Lunch

eTable 2. Students By Grade and Gender, Mean Age By Grade, Race and Ethnicity, and Region

eTable 3. Point Differences Among Genders for American Indian Students Reporting COVID-19–Related Perceived Impacts on Friends, Family, School, and Self

eTable 4. Point Differences Among Genders in American Indian Students Responding Being Worried or Very Worried Over Potential COVID-19 Morbidity and Mortality Outcomes

eTable 5. Differences by Among in American Indian Students Responding About Perceived Changes in COVID-19–Related Psychological Measures

eTable 6. American Indian Students Responding on COVID-19–Related Psychological Measures, by Gender

eReferences.

This supplemental material has been provided by the authors to give readers additional information about their work.

© 2022 Stanley LR et al. *JAMA Network Open.*

eMethods.

1. OYOF Overview.

The data utilized for the primary study were collected during Spring 2021 as part of an ongoing 40-year long epidemiologic study of substance use by American Indian (AI) youth living on or near reservations (Our Youth, Our Future; OYOF). The study is designed to be similar to the on-going Monitoring the Future study (see http://monitoringthefuture.org/) so that comparisons between nationally representative substance use rates and reservation-area AI substance use rates can be made. In addition to substance use rates, the OYOF survey includes items related to risk and protective factors, and for Spring 2021, items related to COVID-19.

2. Sampling Frame.

The OYOF sampling frame was built from three primary sources - the NCES Common Core of Data (CCD), the NCES Private School Universe Survey (PPS), and the Bureau of Indian Education (BIE) National Directory. Only schools with grade 7 were included in the sampling frame as the high schools fed by each sampled middle school were included in the final sample. Criteria used for searching CCD and PPS were: 1) at least 20% AI enrollment and 2) a total enrollment of at least 20 students per grade. Schools from Alaska and Hawaii were excluded. This list was then further refined by including only schools within 25 miles of an AI reservation. These same criteria were used for BIE schools. In the final sampling frame, only schools with an average enrollment by grade (for grades 7 and above) of 20 students were included (when enrollment was known). Grade configurations of the schools included middle/junior high schools (e.g., grades 6-8; 34%), K-8 or similar (35%), 7-12 (11%), K-12 or similar (16%), and other (4%). There are significant numbers of schools with very low enrollment, especially in the Northern Plains and Southwest regions. In the original sampling frame approximately 30.7% of middle schools and 25% of high schools had enrollments below 20 per grade. Due to the very small size of many of these schools (some schools serve fewer than 20 students in total), the exclusion of these schools accounts for significantly less than 10% of students. The sampling frame is updated each year to ensure that schools are included back into the sampling frame if their enrollment has increased. It is worth noting that schools with under 20 students per grade are not surveyed for 2 reasons: 1) the confidentiality of students could be in question in the school report due to the small number of students per grade, and 2) there is a cost associated with surveying each school, as schools are paid to participate, and it takes significant staff resources to survey each school. Regarding the requirement that schools be within 25 miles of a reservation, schools with 20% or more AI enrollment were first identified when constructing the sampling frame. Schools were then deleted that were not near a reservation while noting those that were relatively close to a reservation and thus drew students from the reservation, especially those reservations that do not have a high school. These schools were within 25 miles and thus, this became a parameter for describing our population.

Regions for sampling were determined by first examining regional designations used by the Bureau of Indian Affairs and those set out in a report by Snipp.[1] BIA designations take into account a variety of factors, including culture, geography, and population. Their 12 regions are Northwest, Pacific, Western, Southwest, Rocky Mountain, Great Plains, South Plains, Midwest, Eastern, Navajo, Eastern Oklahoma, and Alaska. Snipp[1] delineated regions based on cultural distinctions: Southwest, Northern Plains, Oklahoma, California, Upper Great Lakes, Alaska, and Southeast, but several regions with reservations were not explicitly included.

After carefully considering cultural distinctions, the number of schools in potential regions, and confidentiality concerns, we designated seven (7) regions – Northwest (which includes northern California reservations; NW), Southwest (which includes Southern California reservations: SW), Northern Plains (NP), Upper Great Lakes (UGL), Southern Great Plains (SGP), Southeast (SE), and Northeast (NE).

The final sampling frame for Spring 2021 contained 318 schools with a grade 7 (not including schools who would be their second year of surveying, as explained below), with the following regional distribution: Northeast: 1.6%; Northern Plains: 31.8%; Northwest 7.2%; Southeast: 1.9%; Southern Great Plains 1.6%; Southwest 41.5%; Upper Great Lakes 14.5%.

3. Sampling and Recruitment Procedures.

Prior to the start of a school year, schools within each region are randomly drawn from the sampling frame such that the regional distribution of sampled schools reflects the regional distribution of AIs ages 10-19 residing on reservations. To calculate the probability of selection for each region, each reservation was assigned its appropriate region, and within each region, the total AI population ages 10-19 was calculated from 2010 Census data. This gives an estimate of AIs attending middle and high school grades on or near reservations within the continental U.S.,

© 2022 Stanley LR et al. *JAMA Network Open.*

although ages are grouped in the Census and do not map exactly onto the ages of 6th-12th grade students. Many tribal and BIE schools do not report enrollments unless taking part in our survey; therefore, we were not able to calculate the AI population by grade from our sampling frame. The Census provides more complete, though still imperfect, data. Because of the small number of schools in the Northeast, Southern Plains, and Southeast, all schools and/or school districts meeting the requirements of the sample are invited to participate in the survey. In other regions, a participation rate of 20% is assumed; thus, we randomly draw approximately 5 times the number of schools needed in the final sample. For the Spring 2021 sample, schools within a region had an equal probability of selection, as enrollments for many schools in the sampling frame were unknown, prior to sampling.

For schools not including high school grades (e.g., middle schools), the high school most likely to be attended by the AI middle school students is determined and added to the drawn sample. Further information about each school included in the drawn sample is gathered, including current enrollments by grade and current contact information. Given the small number of schools within the sampling frame, 30 schools are sought each year to participate in the survey. Schools are asked to participate for 2 consecutive years, as per Monitoring the Future procedures, in order to better identify trends by dampening outliers and/or noise (similar to using moving averages). Fifteen schools were scheduled to repeat from the previous year (2019-2020); however, only 9 agreed to repeat the survey due to the impacts of COVID-19.

With 9 schools surveying for their second year, for Spring 2021 we sought to recruit 21 “new” schools. 318 middle schools were in the sampling frame for new schools, with 163 related high schools. The final draw of schools with a grade 7 was 68 schools. Including the related high school led to a total number of schools of 102, regionally distributed as NE: 6.9%; NP: 36.3%; NW: 11.8%; SE: 4.9%; SW: 26.5%; UGL: 9.8%. Of these, 3 were disqualified due to 100% remote instruction prior to recruitment. (There may have been more schools operating remotely that did not have this information posted on their website or other public area.) Thus, 99 schools were asked to participate.

Recruitment of sampled schools includes a letter of invitation, an email, and a telephone call explaining the project and procedures. Once a school agrees to participate, we obtain all necessary approvals, including school boards and tribal IRBs.

4. Survey Procedures.

For each participating school, the appropriate tribal and school board approvals were obtained. Approximately 3 weeks prior to the scheduled survey, letters were sent to parents of enrolled students in grades 6 and above describing the survey and providing instructions for opting their child out of the survey. This information was also posted on other local media sites, where parents were likely to see it. On the scheduled date, the OYOF survey was administered online to students using Qualtrics software during school hours. School staff read directions prior to survey administration indicating that students could decline to participate or leave blank any questions they did not wish to answer, and these instructions were repeated in the online survey. Eleven (11) schools were operating concurrently (some students remote; some students in-person); 6 schools used a hybrid approach where students were part-time remote and part-time in-person; 3 schools were in-person only. In order to ensure that a staff member was present to read instructions and answer questions, schools were asked to only administer the survey during times when a teacher or other staff member was present, either remotely or in-person. Several schools opted not to survey remote students.

All survey responses were collected anonymously, and all procedures were approved by the university institutional review board. Participating schools received a full report of their survey findings and compensation for resources used to complete the survey process, with mean payment of $1400.

© 2022 Stanley LR et al. *JAMA Network Open.*

5. Spring 2021 Sample

Eleven “new” schools agreed to participate (11.1% participation rate) in the Spring 2021 survey. (Three schools responded positively to recruitment, but had to be excluded due to 100% remote instruction.) With the 9 schools in their second year of surveying, the total participation rate among schools was 17.1% (20/117). (Spring 2021 was a particularly difficult time to recruit due to COVID-19 protocols, especially those on reservations.) The distribution of participating schools compared to those schools that were in the draw but did not participate are shown in eTable 1.

© 2022 Stanley LR et al. *JAMA Network Open.*

eTable 1. Comparison of Participating Schools and Nonparticipating Schools in Spring 2021 Sample, By Type Of School, Location, Ethnicity Composition, and Percentage of Students Receiving Free Lunch[a]

Number of schools (%)	Surveyed	Non-Participating
Type of School		
Private	0 (0)	1 (1.0)
Public	19 (95.0)	71 (73.2)
BIE/Tribal	1 (5.0)	25 (25.8)
On or Off Reservation		
On	11 (55.0)	62 (63.9)
Off	9 (45.0)	35 (36.1)
Level of Rurality[b]		
Suburban, large	0 (0.0)	1 (1.0)
Town, fringe	0 (0.0)	2 (2.1)
Town, distant	6 (30.0)	5 (5.2)
Town, remote	2 (10.0%)	4 (4.1)
Rural, fringe	5 (25.0)	12 (12.4)
Rural, distant	1 (5.0)	18 (18.6)
Rural, remote	6 (30.0)	44 (45.4)
Not available	0 (0.0)	11 (11.3)
Total Enrollment[c]		
20-49	0 (0.0)	1 (1.0)
50-149	6 (30.0)	39 (40.2)
150-249	6 (30.0)	19 (19.6)
250-499	5 (25.0)	24 (24.7)
500-749	2 (10.0)	9 (9.3)
750+	1 (5.0)	5 (5.2)
% of enrollment that is American Indian[b]		
<25	3 (15.0)	13 (14.3)
25-49.9	3 (15.0)	10 (11.0)
50-74.9	7 (35.0)	16 (17.6)
75-94.9	3 (15.0)	11 (12.1)
95-100	4 (20.0)	41 (45.1)
Not available	0 (0.0)	6 (6.2)
% of students with free lunch[b]		
<50	7 (35.0)	17 (17.5)
50-89.9	6 (30.0)	28 (28.9)
90-100	7 (35.0)	47 (48.5)
Not available	0 (0.0)	5 (5.5)

[a]Surveyed and non-surveyed schools include both “new” schools and those schools that are repeats from the previous year.
[b]Data comes from Common Core of Data for public and private schools. https://nces.ed.gov/datatools/
[c]Data comes from Common Core of Data for public and private schools and personal correspondence with individual schools.

© 2022 Stanley LR et al. *JAMA Network Open.*

6. Description of Measures.

COVID-19 morbidity and mortality. To measure self and family members or close friends COVID-19 morbidity and mortality, we used measures from CASPE Adolescent Self-Report Survey[2] (hereafter CASPE) for COVID-19 self-reported diagnoses, hospitalizations, and deaths. Percentage of students reporting being tested was obtained from the COVID-19 self-reported diagnosis question.

Social impacts. Changes in family relationships, friend relationships, school engagement, and social isolation were measured using a 4-point scale from "not true at all" to "very true." Four items measured changes in family time, closeness, conflict, and monitoring while four items measured impacts on relationships with friends (e.g., closeness, method of engagement). Ten items measured school engagement impacts (e.g., changes in grades, attendance, enjoyment) as well as lack of resources at home for schoolwork. Overall impacts on social connection were measured with two items - time spent alone and a separate question of feeling of connectedness with others (from Environmental Influences on Child Health Outcomes COVID-19 Questionnaire-Child Self-Report[3] (hereafter, C19-cPV)), measured on a 5-point scale from "much less socially connected" to "much more socially connected." Items can be found in Table 2.

Changes in psychological measures. Frequency of (5-point scale of "very often" to "never") and changes in (5-point scale of "much less" to "much more") experiencing anxiety, depression, sadness, and other emotions and thoughts since COVID-19 began were measured using items from the 2021 Monitoring the Future survey (Richard Miech, PhD, email correspondence, 10/14/2020). Worry over COVID-19 related outcomes (e.g., catching COVID-19) were measured using adapted items from CASPE and the Coronavirus Health Impact Survey (CRISIS), V0.3.[4]

Sex. The OYOF survey uses wording from the Monitoring the Future survey to measure sex, asking "How do you describe yourself?, with responses of Male (M), Female (F), and Another (A). We did not ask about gender identity.

7. Analysis.

<u>Sample weights</u>

All students in grades 6-12 are eligible to be surveyed, and weights are computed based on student enrollments and participation. Weights are computed as follows.

$W_{g,h}$ = (# of reservation-area AI students in grade *g* within region *h*)/(# of reservation-area AI students enrolled in sampled schools in grade *g* within region *h*),

where the numerator is estimated from Census 2010 American Indians of one race or more than one race ages 10-19 for all reservations within a region and the denominator is calculated after obtaining enrollments from participating schools. Non-response weights are calculated as:

$W_{i|g|j,}$ =(# of AI students enrolled in grade *g* in school *j*)/(# of AI students completing survey in grade *g*, school *j*)

The final weight for a student i in grade g in school j which is located in region h is:

$$W_{i|g|j,\,h} = W_{g,h} * W_{i|g|j}$$

<u>Proportion and confidence interval estimation</u>

For each item discussed above, frequency of outcomes (95% CI) were computed using survey commands of Stata® statistical software, designating stratification by region and school as the primary sampling unit. The survey commands for proportion with default linearized variance estimation were used to calculate proportions and 95% CIs. An example command is: svy linearized: proportion CVp25_b1r, over(sex). Differences by grade group (6-8 and 9-12) and sex were tested using Pearson chi-square tests adjusted for complex samples while point differences in proportions between sexes and their confidence intervals were calculated using lincom commands in Stata.

8. Participants.

In total, 3847 students in grades 6-12 were surveyed, with this study using a subsample of students self-reporting as AI (N=2559). Participants represented 60.4% of eligible students in these schools, with 70% of schools

© 2022 Stanley LR et al. *JAMA Network Open.*

surveying 60% or more of their eligible students. Fewer than 1% of students across participating schools were opted out of participation in OYOF by their parents, and the primary reason that students did not take the survey was due to absence on the day of survey administration. During COVID-19, AI schools had high absentee rates among students, and students not attending school may have had limited access to technology needed for remote learning.[5] This may have introduced bias in this study's findings. A question on the survey asked students in participating schools whether they were taking the survey remotely or in-person. A comparison of responses presented in this study found no significant differences in responses by where the survey was taken (remotely or in-person), lending some credence to remote students not being different from in-person students. However, these remote students may have been more likely to have technology access, thus affecting their responses to school questions in Table 2 and potentially other responses. Finally, the exact number of students who refused to take the survey on the survey administration date (that is, those who were present but refused to complete the survey) cannot be determined because there is no record in the database for students who choose not to open the survey link. Because the survey is anonymous, we ask teachers to be in an area of the classroom or lab where they cannot observe student responses.

Sample sizes by sex and mean age, per grade, are presented in eTable 2, in addition to race/ethnicity and region. It is worth noting that Spring 2021 was a challenging time for surveying (no surveying occurred Spring 2020 or Fall 2020 due to COVID-19), and thus, there may be bias in our data due to a variety of factors that are specific to the pandemic. At the same time, there is no other broad-scale survey of reservation-area AI adolescents of this type. Therefore, even with its limitations, the data can make a positive contribution to understanding COVID-19's "impacts" on this unique population.

**eTable 2.** Students By Grade and Gender, Mean Age By Grade, Race and Ethnicity, and Region					
Grade	Total	Sex[a]			Mean age (years)
		Female	Male	Another	
6	268	129 (48.1)	138 (51.5)	0 (0.0)	11.7
7	413	196 (47.5)	193 (46.7)	24 (5.8)	12.7
8	398	191 (48.0)	186 (46.7)	18 (4.5)	13.8
9	507	257 (50.7)	236 (46.5)	14 (2.8)	14.8
10	367	192 (52.3)	168 (45.8)	7 (1.9)	15.8
11	316	165 (52.2)	147 (46.5)	4 (1.3)	16.8
12	290	154 (53.1)	133 (45.9)	3 (1.0)	17.5
6-12 grades	2559	1284 (50.2)	1201 (46.9)	70 (2.7)	14.7
Race/ethnicity[b]					N (%)
AI only					1574 (61.5)
AI plus at least one of the following:					
Alaska Native					53 (2.1)
Asian American					28 (1.1)
Black					180 (7.0)
Hawaiian/Pacific Islander					31 (1.2)
Hispanic/Latino					271 (10.6)
White					833 (32.6)
Region					
Northeast					90 (3.5)
Northern Plains					207 (8.1)
Northwest					84 (3.3)
Southeast					668 (26.1)
Southern Plains					277 (10.8)
Southwest					1005 (39.3)
Upper Great Lakes					228 (8.9)

[a]Numbers of female, male and another may not add to the total due to missing values.
[b]Respondents could select multiple race/ethnicities.

© 2022 Stanley LR et al. *JAMA Network Open.*

eTable 3. Point Differences Among Genders for American Indian Students Reporting COVID-19–Related Perceived Impacts on Friends, Family, School, and Self				
		Difference between % reporting somewhat or very true (95% CI)		
	N[a]	Male - Female	Male - Another	Female - Another
**Impacts on family relationships**				
I spend more time with my family.	1479	2.1 (-4.5, 8.6)	9.3 (-15.8, 34.4)	7.2 (-18.2, 32.7)
My family has more conflict.	1448	-1.6 (-6.6, 3.4)	-7.0 (-27.4, 13.4)	-5.4 (-26.9, 16.2)
My family is closer.	1463	0.1 (-8.6, 10.6)	12.0 (-12.3, 36.2)	11.0 (-18.0, 39.9)
My parents/guardians supervise my activities more.	1453	-3.8 (-10.0, 2.4)	-10.4 (-41.9, 21.1)	-6.6 (-34.3, 21.2)
**Impacts on relationships with friends**				
I see my friends more remotely or online (texting, social media).	1475	-5.4 (-14.0, 3.2)	-13.0 (-36.8, 10.7)	-7.6 (-35.5, 20.2)
I see my friends more in person.	1480	7.3 (1.9, 12.6)[c]	10.5 (-6.9, 27.8)	3.2 (-13.3,19.8)
I'm worried that I will lose friends because I can't see them as often.	1475	-7.7 (-11.7, -3.7)[d]	-29.3 (-41.8, -16.8)[d]	-21.6 (-36.6, -6.6)[d]
I feel closer to my friends.	1490	9.7 (5.0, 14.4)[d]	1.7 (-11.4, 14.8)	-8.0 (21.0, 4.9)
**Impacts on school**				
I am getting better grades in school.	1457	6.6 (2.1, 11.1)[d]	6.9 (-19.8, 33.6)	0.3 (-25.1, 25.8)
I am enjoying school more.	1475	6.3 (-1.4, 14.0)	-6.5 (-19.0, 5.9)	-12.8 (-27.5, 1.8)
It is harder for me to focus on my schoolwork.	1474	-12.0 (-20.6, -3.3)[c]	-26.3 (-45.3, -7.3)[c]	-14.4 (-34.1, 5.3)
I am falling behind in my schoolwork more.	1458	-11.3 (-22.6, -0.0)[c]	-24.2 (-38.8, -9.6)[d]	-12.9 (-35.0, 9.3)
I attend school more (either remotely or in person).	1456	-1.0 (-5.1, 3.1)	1.3 (-13.3, 16.0)	2.4 (-14.5, 19.2)
I am more worried about school.	1223	-8.6 (-14.0, -3.3)[d]	-17.0 (-33.7, -0.2)[c]	-8.3 (-23.3, 6.6)
I miss participating in or attending school events.	1465	-12.0 (-17.1, -7.0)[d]	-9.6 (-31.0, 11.8)	2.4 (-16.8, 21.6)
I don't have the technology I need at home to complete my schoolwork (computer, wifi/internet).	1438	-1.8 (-6.4, 2.8)	0.2 (-18.7, 19.0)	2.0 (-17.2, 21.2)
I don't have the space I need at home to complete my schoolwork (a desk to work on, a quiet room).	1435	-9.9 (-16.7, -3.1)[d]	-21.2 (-36.7, -5.8)[d]	-1.3 (-28.3, 5.7)
It is difficult to concentrate on my schoolwork because of what else is happening at home.	1434	-2.2 (-9.8, 5.2)	-12.7 (-28.9, 3.5)	-10.4 (-26.6, 5.8)
**Impacts on social connection**				
I spend more time alone.	1453	-15.2 (-21.8, -8.5)[d]	-45.7 (-54.5, -36.9)[d]	-30.5 (-40.1, -20.9)[d]
I feel less socially connected to people.[b]	2456	-13.2 (-15.3, -11.)[d]	-3.9 (-13.8, 5.9)	9.2 (-1.7, 19.6)

[a]Sample sizes for all but the last question in this table are significantly less than 2559 partially due to the planned missingness design and partially due to students not responding to the question. For all questions but the last one, the sample sizes by sex averaged 722 for females, 668 for males, and 39 for another sex.
[b]This question was based on a 5-point scale of much less socially connected, less socially connected, about the same, more socially connected, and much more socially connected. Percentages indicate those who answered they felt much less or less socially connected. The larger N is due to this question not being part of the planned missing design so all students could answer it. Also, it was near the start of the survey and thus had a lower percentage of missingness due to survey attrition.
[c]Estimate is significantly different from zero at $p<.05$.
[d]Estimate is significantly different from zero at $p<.01$.

© 2022 Stanley LR et al. *JAMA Network Open.*

eTable 4. Point Differences Among Genders in American Indian Students Responding Being Worried or Very Worried Over Potential COVID-19 Morbidity and Mortality Outcomes

How worried are you about…	N[a]	Male - Female	Male - Another	Female - Another
…getting COVID-19?	1538	-5.5 (-14.9, 3.9)	2.7 (-8.4, 13.7)	8.1 (2.3, 14.0)[b]
… a family member getting COVID-19?	1559	-14.8 (-19.6, -10.0)[c]	-2.0 (-18.9, 14.8)	12.8 (-3.0, 28.5)
…dying from COVID-19?	1528	-10.0 (-18.4, -1.6)[b]	19.3 (9.5, 29.2)[c]	29.3 (17.9, 40.7)[c]
…a family member dying from COVID-19?	1528	-7.6 (-12.6, -2.6)[c]	1.4 (-15.2, 18.0)	8.9 (-8.9, 26.8)
…giving someone else COVID-19?	1556	-11.0 (-15.3, -6.7)[c]	-2.4 (-16.9, 12.2)	8.6 (-7.2, 24.5)

[a]Sample sizes in this table are significantly less than 2559 partially due to the planned missingness design and partially due to students not responding to the question. For all questions, the sample sizes by sex averaged 785 for females, 719 for males, and 39 for another sex.
[b]Estimate is significantly different from zero at $p<.05$.
[c]Estimate is significantly different from zero at $p<.01$.

© 2022 Stanley LR et al. *JAMA Network Open.*

eTable 5. Differences by Among in American Indian Students Responding About Perceived Changes in COVID-19–Related Psychological Measures[a]

	Difference by sex of % experiencing more or much more than before COVID-19 (95% CI)			Difference by sex of % experiencing about the same as before COVID-19 (95% CI)			Difference by sex of % experiencing less or much less than before COVID-19 (95% CI)		
Compared to before COVID-19, are you…	Male - Female	Male- Another	Female - Another	Male - Female	Male- Another	Female - Another	Male - Female	Male- Another	Female - Another
More or less sad?	-16.9[d] (-3.0, -10.8)	-39.8[d] (-67.8, -11.8)	-22.9 (-52.6, 6.7)	2.0 (-4.6, 8.5)	9.2 (16.6, 35.1)	7.3 (-12.9, 27.4)	14.9[d] (5.9, 24.0)	30.6[d] (21.5, 39.7)	15.7[c] (3.6, 27.7)
More or less lonely?	-14.6[d] (-22.0, -7.3)	-34.1[c] (-61.6, -7.6)	-19.4 (-46.8, 7.9)	0.9 (-6.5, 8.3)	4.5 (-11.0, 20.0)	3.6 (-14.0, 21.2)	13.8[d] (6.9, 20.6)	29.6[d] (11.5, 47.7)	15.8 (-3.7, 35.3)
More or less depressed?	-15.7[d] (-20.0, -11.5)	-36.6[d] (-56.0, -17.2)	-20.9[c] (-38.7, -3.0)	1.0 (-7.0, 9.1)	0.0 (-17.7, 17.7)	-1.0 (-17.5, 15.5)	14.7[d] (6.3, 23.1)	36.6[d] (25.1, 48.0)	21.9[d] (13.8, 30.0)
More or less angry?[b]	-15.4[d] (-25.7, -5.2)	-22.8 (-47.5, 1.9)	-7.4 (-27.2, 12.4)	0.0 (-12.5, 12.8)	3.5 (-26.8, 33.8)	3.4 (-20.2, 26.9)	15.3[d] (6.8, 23.7)	19.3[d] (5.6, 33.0)	4.0 (-14.4, 22.5)
More or less worried?	-9.8[d] (-14.6, -4.9)	-17.9[d] (-28.1, -7.7)	-8.1 (-19.3, 3.1)	-3.6 (-7.8, 0.6)	-1.2 (-14.0, 16.4)	4.8 (-12.2, 28.8)	13.4[d] (8.9, 17.9)	16.7[d] (4.9, 28.4)	3.3 (-11.9, 18.5)
More or less anxious?	-17.5[d] (-24.7, -10.3)	-43.3[d] (-59.6, -27.0)	-25.8[d] (-41.7, -9.9)	0.2 (-7.5, 7.9)	8.9 (-5.9, 23.7)	8.7 (-4.9, 22.3)	17.3[d] (12.6, 22.0)	34.4[d] (24.0, 44.9)	17.1[d] (7.9, 26.4)
Having trouble sleeping?[b]	-13.7[d] (-17.7, -9.7)	-38.2[d] (-51.2, -25.2)	-24.4[d] (-37.1, -11.8)	0.5 (-6.0, 7.0)	6.9 (-5.7, 19.5)	6.4 (-5.0, 17.8)	13.2[c] (5.8, 20.7)	31.3[d] (23.3, 39.3)	18.1[d] (10.2, 26.0)
Interested in normal activities?	-3.8 (-11.0, 3.4)	-28.1[c] (-55.1, -1.1)	-24.3[c] (-47.2, -1.5)	-4.9 (-13.6, 3.7)	17.2 (0.5, 33.9)	22.1 (9.5, 34.7)	8.7[c] (1.5, 16.0)	10.9 (-5.5, 27.4)	2.2 (-12.9, 17.4)
Having trouble concentrating?	-9.4[d] (-14.7, -4.2)	-29.9[d] (-47.6, -12.2)	-20.5[c] (-37.0, -4.0)	3.4 (-3.5, 10.7)	13.4 (-7.1, 33.9)	9.8 (-7.6, 27.3)	5.8[c] (0.8, 10.9)	16.5[d] (8.6, 24.5)	10.7[c] (2.0, 19.4)

[a]Number of observations for each item varied from 1503 to 1534. Sample sizes are significantly less than 2559 partially due to the planned missingness design and partially due to students not responding to the question. For all questions, the sample sizes by sex averaged 767 for females, 701 for males, and 39 for another sex.
[b]6th – 8th grade students were more likely to report feeling more or much more this way. For students in grades 6-8, 33.1% (95% CI, 30.2-36.1) of students felt more angry and 43.5% (95% CI, 38.6-48.5) had more trouble sleeping. For students in grades 9-12, 27.7% (95% CI, 25.5-30.0) felt more angry and 34.9% (95% CI, 29.8-40.5) had more trouble sleeping.
[c]Estimate is significantly different from zero at $p<.05$.
[d]Estimate is significantly different from zero at $p<.01$.

© 2022 Stanley LR et al. *JAMA Network Open.*

**eTable 6.** American Indian Students Responding on COVID-19–Related Psychological Measures, by Gender[a]			
	Percentage (%) responding often or very often (95% CI)		
Since the start of COVID-19, how often have you…	Female	Male	Another
Felt sad?	61.9 (56.7, 66.8)[c]	34.0 (29.5, 38.8)[g]	86.5 (59.3, 96.6)
Felt lonely?	59.0 (45.2, 63.6)[c,d]	34.7 (27.7, 42.4)[g]	84.4 (65.7, 93.9)
Felt depressed?	50.2 (41.8, 58.6)[c,e]	21.6 (18.2, 25.4)[g]	80.9 (64.0, 91.0)
Felt angry?	46.2 (41.0, 51.5)[c]	31.0 (26.5, 35.8)[f]	48.6 (35.4, 62.0)
Felt worried?	52.3 (47.0, 57.5)[c]	35.4 (31.2, 39.8)[f]	61.9 (34.0, 83.6)
Felt anxious?	48.7 (40.5, 57.0)[c,e]	21.3 (18.0, 25.1)[g]	76.7 (58.3, 88.6)
Had trouble sleeping?	61.5 (56.6, 66.1)[c,e]	33.6 (27.2, 40.7)[g]	92.2 (68.5, 98.5)
Felt less interested in normal activities?	53.9 (50.2, 57.6)[c,e]	30.8 (24.0, 38.5)[g]	82.0 (62.5, 92.6)
Had trouble concentrating?	64.0 (57.7, 69.8)[c]	40.8 (36.6, 45.2)[g]	79.8 (53.5, 93.1)

[a]Number of observations for each item varied from 1503 to 1534.
[b]Female and male estimates differ at $p<.05$.
[c]Female and male estimates differ at $p<.01$.
[d]Female and another estimates differ at $p<.05$.
[e]Female and another estimates differ at $p<.01$.
[f]Male and another estimates differ at $p<.05$.
[g]Male and another estimates differ at $p<.01$.

© 2022 Stanley LR et al. *JAMA Network Open.*

eReferences

1. Snipp CM. The size and distribution of the American Indian population: Fertility, mortality, migration, and residence. *Population Research & Policy Review.* 1997;16:61-93.
2. Ladouceur CD. COVID-19 Adolescent Symptom & Psychological Experience Questionnaire (CASPE). 2020; https://www.nlm.nih.gov/dr2/CASPE_AdolSelfReport_Qualtrics.pdf.
3. Environmental Influences on Child Health Outcomes. COVID-19 Questionnaire - Child Self-Report Primary Version. 2020; https://www.nlm.nih.gov/dr2/C19-cPV_COVID-19_Questionnaire-Child_Self-Report_Primary_Version_20200409_v01.30.pdf.
4. Child Mind Institute. The Coronavirus Health Impact Survey (CRISIS) V0.3, Youth Self-Report Baseline Form. 2020; https://www.nlm.nih.gov/dr2/CRISIS_Youth_Self-Report_Baseline_Current_Form_V0.3.pdf.
5. Tsethlikai M, Sarche M, Barnes JV, Fitzgerald H. Addressing Inequities in Education: Considerations for American Indian and Alaska Native Children and Youth in the Era of COVID-19. 2020; https://www.srcd.org/research/addressing-inequities-education-considerations-american-indian-and-alaska-native-children. Accessed 12/28/2021, 2021.

© 2022 Stanley LR et al. *JAMA Network Open.*
